# The clinical promise of biomarkers of synapse damage or loss in Alzheimer’s disease

**DOI:** 10.1186/s13195-020-00588-4

**Published:** 2020-03-02

**Authors:** Martí Colom-Cadena, Tara Spires-Jones, Henrik Zetterberg, Kaj Blennow, Anthony Caggiano, Steven T. DeKosky, Howard Fillit, John E. Harrison, Lon S. Schneider, Phillip Scheltens, Willem de Haan, Michael Grundman, Christopher H. van Dyck, Nicholas J. Izzo, Susan M. Catalano, the Synaptic Health Endpoints Working Group

**Affiliations:** 1 Centre for Discovery Brain Sciences, UK Dementia Research Institute at The University of Edinburgh, Edinburgh, UK; 2 Department of Psychiatry and Neurochemistry, University of Gothenburg, Mölndal, Sweden; 3 Clinical Neurochemistry Laboratory, Sahlgrenska University Hospital, Mölndal, Sweden; 4 Department of Neurodegenerative Disease, UCL Institute of Neurology, London, UK; 5 UK Dementia Research Institute at UCL, London, UK; 6 Cognition Therapeutics. Inc., Pittsburgh, PA USA; 7 McKnight Brain Institute, University of Florida, Gainesville, FL USA; 8 Alzheimer’s Drug Discovery Foundation, New York, NY USA; 9 Metis Cognition Ltd, Kilmington, UK; 10 Alzheimer Center, AUmc, Amsterdam, The Netherlands; 11 Institute of Psychiatry, Psychology & Neuroscience, King’s College London, London, UK; 12 Keck School of Medicine of USC, Los Angeles, CA USA; 13 Alzheimer Center Amsterdam, Department of Neurology, Amsterdam Neuroscience, Vrije Universiteit Amsterdam, Amsterdam UMC, Amsterdam, The Netherlands; 14 Department of Clinical Neurophysiology and MEG, VU University Medical Center, Amsterdam, Netherlands; 15 Global R&D Partners, San Diego, CA USA; 16 Alzheimer’s Disease Research Unit and Departments of Psychiatry, Neurology, and Neuroscience, Yale School of Medicine, New Haven, CT USA

**Keywords:** Alzheimer’s disease, Synapse, Biomarker, Cerebrospinal fluid, Positron emission tomography, Electroencephalography

## Abstract

**Background:**

Synapse damage and loss are fundamental to the pathophysiology of Alzheimer’s disease (AD) and lead to reduced cognitive function. The goal of this review is to address the challenges of forging new clinical development approaches for AD therapeutics that can demonstrate reduction of synapse damage or loss.

The key points of this review include the following:

- Synapse loss is a downstream effect of amyloidosis, tauopathy, inflammation, and other mechanisms occurring in AD.
- Synapse loss correlates most strongly with cognitive decline in AD because synaptic function underlies cognitive performance.
- Compounds that halt or reduce synapse damage or loss have a strong rationale as treatments of AD.
- Biomarkers that measure synapse degeneration or loss in patients will facilitate clinical development of such drugs.
- The ability of methods to sensitively measure synapse density in the brain of a living patient through synaptic vesicle glycoprotein 2A (SV2A) positron emission tomography (PET) imaging, concentrations of synaptic proteins (e.g., neurogranin or synaptotagmin) in the cerebrospinal fluid (CSF), or functional imaging techniques such as quantitative electroencephalography (qEEG) provides a compelling case to use these types of measurements as biomarkers that quantify synapse damage or loss in clinical trials in AD.

**Conclusion:**

A number of emerging biomarkers are able to measure synapse injury and loss in the brain and may correlate with cognitive function in AD. These biomarkers hold promise both for use in diagnostics and in the measurement of therapeutic successes.

## Background

Alzheimer’s disease (AD) and related dementias afflict nearly 44 million people worldwide [1]. In the USA, nearly 6 million people have AD, a number that is expected to double by 2050 [2]. Only symptomatic treatments are currently available, and disease modeling techniques suggest that the beneficial effects of current treatments may peak by 6 months [3, 4]. More effective symptomatic treatments or first-of-a-kind disease-modifying therapies for AD continue to be a huge unmet medical need; these treatments would significantly impact the quality of life annual healthcare expenditure for AD patients, which were estimated to be $277B annually in 2018 and up to $1100B annually by 2050 [2].

Hypotheses regarding etiology of AD and potential targets for pharmacologic intervention have evolved over the recent decades of intense industry and academic research. Neurotransmitter hypotheses, while giving rise to the first drugs approved for treating AD, generated means for symptomatic relief but failed to generate disease-altering treatments [5]. Amyloid plaque- and tau tangle-related hypotheses, focused on aggregated Aβ peptide and tau protein, appeared to offer promising targets for disease-altering therapies, but most clinical programs targeting Aβ generation with small molecules and Aβ clearance with antibodies have been disappointing [6, 7]. Treatment with several anti-Aβ antibodies (solanezumab, with a high affinity for monomeric Aβ, and aducanumab and BAN2401, which target fibrillar Aβ) was associated with a small slowing of cognitive decline in subsets of patients with AD, but those targeting fibrils are associated with vasogenic edema and cerebral microhemorrhages, possibly limiting their clinical usefulness [7]. Understanding the role of soluble Aβ aggregates has led to the new hypotheses that these Aβ oligomers may be responsible for the neurotoxic etiology of AD, with hopes that therapeutics that reduce their synaptotoxicity may delay or stop the progression of AD [8]. Monitoring treatment-related reduction of such toxicity may provide suitable biomarker endpoints for drug efficacy and is independent of etiology of disease.

A foundational principle of neuroscience is that synaptic function underlies cognition. There is widespread acceptance of the premise that synapse damage or loss is the objective sign of neurodegeneration that is most highly correlated with cognitive decline in AD; this is supported by clinical, post-mortem, and non-clinical evidence as summarized below. Objective measures of synaptic damage or loss are therefore a special category of biomarkers expected to be most closely correlated with cognitive function.

The goal of this paper is to review the concept of biomarkers of synapse damage as a potential approvable endpoint for treatment in AD and other neurological indications and to review the literature in order to assist biopharmaceutical drug developers and regulators in addressing the challenges of forging new pathways for the approval of synaptoprotective AD therapeutics. The first portion of this manuscript will review the critical role played by synaptic damage in the pathophysiologic processes that underlie AD and their relation to cognitive decline. The second portion will review currently available biomarkers that measure synapse damage or loss in living patients, with a view towards their use as surrogate endpoints in clinical trials in AD.

## The roles of synaptic damage and loss in cognition

The idea that changes in synapses mediate information storage dates back to Santiago Ramon y Cajal’s anatomical observations of brain structure in the late 1890s [9]. This gained popularity in the mid-twentieth century with Hebb’s postulate that synapses between neurons will be strengthened if they are active at the same time, and that this process contributes to learning [10]. This was supported experimentally by Kandel’s studies in *Aplysia* [11]. This concept was underscored by the discoveries of synaptic long-term potentiation by Bliss and Lomo [12] and the hippocampal synaptic plasticity in memory formation by Morris and colleagues [13]. In recognition of the importance of synaptic function to cognition, awards including the Brain Prize and the Nobel Prize have been awarded to multiple scientists for their work in this field.

Synapse dysfunction and loss correlates most strongly with the pathological cognitive decline experienced in Alzheimer’s disease [14–19]. This association was initially described through two independent methods, the estimation of synapse number using electron microscopy techniques [16] and measurements of synaptic protein concentrations [19], each of which showed a strong correlation between synapse number (or synaptic proteins) and cognitive scores on the Mini-Mental Status Examination (MMSE). This concept has been robustly replicated using a variety of approaches [14, 18, 20–26], including disease models. While the molecular cascades leading to synapse degeneration in AD have yet to be fully determined, there is ample evidence from both human brain and disease models supporting synaptotoxic roles of soluble pathological forms of Aβ and tau, as well as glial-mediated neuroinflammation (see [14] for an excellent recent meta-analysis). This paper will review evidence of these mechanisms, as well as approaches for their detection in patients.

## Mechanisms of synapse damage and loss in AD

**Fig. 1:**
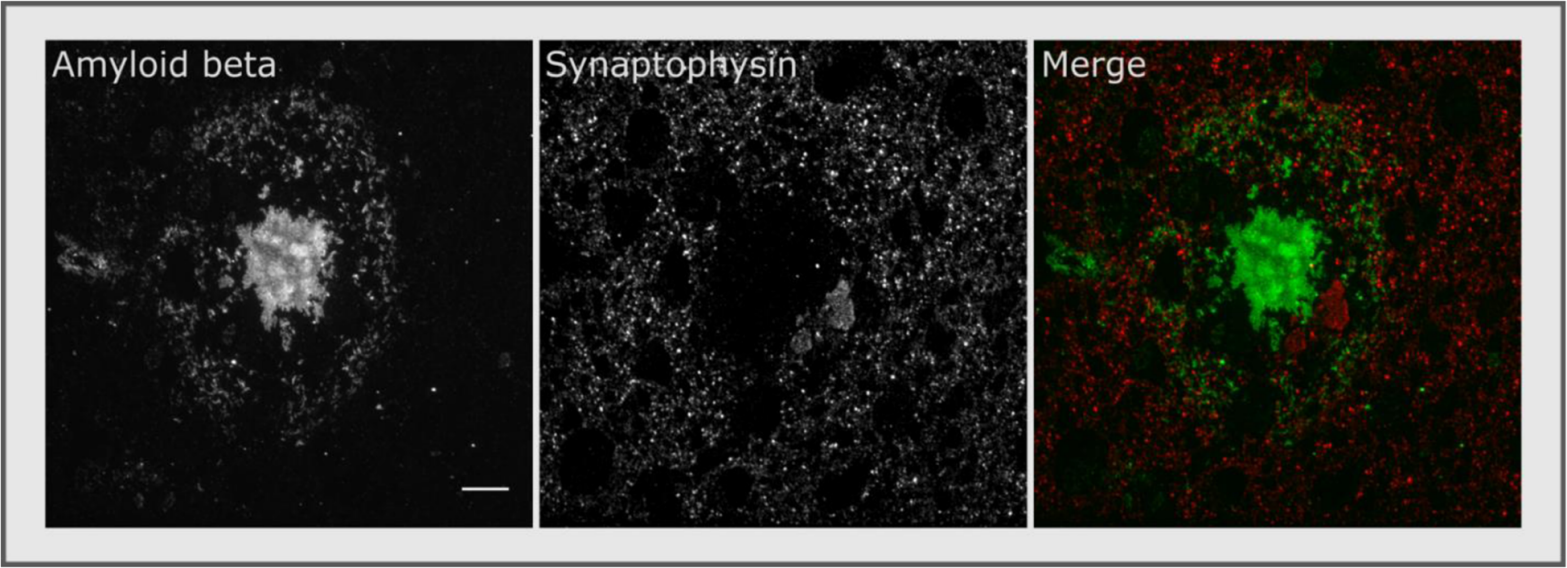
High-resolution array tomography imaging reveals plaque-associated synapse loss in human temporal cortex. Scale bar 10 μm

Amyloid plaques formed of aggregated Aβ peptide are one of the defining pathological lesions of AD [27–29]. In both human brain and mouse models expressing familial AD-associated amyloid precursor protein and presenilin mutations, plaques are associated with local synapse loss [Fig. 1, [30–34]] as well as memory and synaptic plasticity deficits [35–37]. However, total plaque load is not the factor most strongly correlated with cognitive decline [38] or synaptic pathology [17, 39] in AD. Instead, abundant data demonstrate that soluble forms of Aβ, rather than the large insoluble fibrils in plaques, are toxic to synapses [15, 40]. Lambert and colleagues found that fibril-free synthetic forms of Aβ oligomers (AβO) inhibited long-term potentiation (LTP) ex vivo [41], and in 2002, Walsh and colleagues demonstrated that naturally secreted AβO disrupt LTP in vivo [42]. Since then, many studies have shown that AβO may drive the cognitive impairment found in animal models of AD [43–45] and potentially also in human AD [46–48].

Exposure to oligomers in vitro produces rapid reduction in the expression of many synaptic proteins required for normal neurotransmission and for learning and memory formation within hours [49]; longer exposure produces frank loss of synapses and spines [45, 49–51]. Higher, non-physiological concentrations result in rapid neuronal cell death.

The presence of AβO has been correlated with synaptic plasticity impairment and frank synapse loss in mice and cell models [45, 49–51] and in human brains in AD [30, 52, 53]. Furthermore, AβO have been visualized within individual synapses of both mouse models and AD cases using high-resolution imaging techniques [30, 31, 54], arguing strongly that they may directly contribute to synaptic and cognitive dysfunction.

While Aβ monomers may interact with many receptors, in model systems, AβO have been demonstrated to bind to synaptic receptors including cellular prion protein, NgR1, EphB2, and PirB/LilrB2; additional receptor proteins have yet to be rigorously defined [55–61]. One important regulator of the oligomer receptor complex is the sigma-2 protein receptor complex [62, 63], the target of the AD disease-modifying drug candidate CT1812 [64]. Downstream of interacting with synaptic receptors, robust evidence suggests AβO cause calcium influx and downstream synaptic dysfunction [15, 65, 66].

Another defining neuropathological lesion of AD is the aggregation of truncated, misfolded, and hyperphosphorylated tau into neurofibrillary tangles [27]. Tau pathology correlates with neuron loss and cognitive decline in AD [28, 67]. In accordance with the observation that tau causes neuron death, mouse models that express tau mutations that cause frontotemporal dementias with tau pathology demonstrate neuron loss [68–71], early synapse loss, and disruption of neuronal network function [72–77]. As has been observed with Aβ, the forms of tau that may be toxic are the soluble, non-fibrillar, and highly reactive forms, the oligomers [78–80].

Loss of physiological tau function may contribute to synapse degeneration by impairing axonal transport of cargoes needed at synapses, including mitochondria [81, 82]. Part of the synaptic and network dysfunction in tauopathy mice and in AD is likely due to direct effects of tau at synapses. Along with the canonical microtubule stabilizing role of tau, this versatile protein has also been shown to play a physiological role in dendrites including post-synaptic densities and in pre-synaptic terminals [83–85]. In human AD brain, small aggregates of phospho-tau are observed in both pre-synaptic and post-synaptic regions, and several groups have observed phospho-tau in biochemically isolated synaptic fractions [85–87]. Importantly, accumulation of phospho-tau in synaptic fractions was much higher in people with AD (cases) than in people with high pathological burdens who did not exhibit dementia symptoms [48]. Together, these data strongly indicate that pathological forms of tau at synapses contribute to synaptic dysfunction.

Based on the genetic causes of rare forms of familial AD, which all act to increase Aβ accumulation, and the timing of pathological development where plaque pathology is an early pathological feature preceding appreciable tau pathology by many years, it is widely thought that Aβ is “upstream” of tau in initiating AD pathogenesis [88]. One of the key challenges in this field is understanding the links between Aβ and tau, and recent data indicate that these proteins may cooperate to cause synaptic degeneration. Several pathways involving tau have been implicated in AβO-mediated synapse loss. AβO activation of the NMDA receptor has been reported to cause excitotoxicity through the recruitment of Fyn kinase by tau to the post-synaptic density in mice [83, 89, 90]. Lowering tau levels also protects against some of the synaptic effects of AβO [91, 92].

Beyond the direct effects of these pathological proteins on neurons and synapses, epidemiologic and genetic data strongly implicate inflammatory mechanisms in synapse damage in AD. In particular, recent data indicate that microglia may play an active role in synapse loss [93]. The most important genetic risk factor for late-onset AD is inheritance of the apolipoprotein E epsilon 4 (*APOE* ε4) allele [94]. The ApoE4 isoform is highly expressed in astrocytes under physiological conditions, but its expression is upregulated in microglia in mouse models of AD [95]. The effects of AβO at synapses are exacerbated by ApoE4 in plaque-bearing mouse models and human AD brain and are ameliorated by removing endogenous ApoE [30, 96, 97]. Triggering receptor expressed on myeloid cells 2 (TREM2), complement receptor 1 (CR1), and CD33 are all expressed in microglia, where they may affect phagocytosis of synapses [93]. The complement system has emerged recently as particularly interesting in AD because the tagging of synapses with C1q downstream of both Aβ and tau pathology causes CR3-mediated microglial phagocytosis of synapses [98–102]. While several members of the complement pathway have been observed to be upregulated in AD brain and to correlate with tau pathology [101, 102], it remains unknown whether microglial phagocytosis of synapses in human disease actively drives synapse loss or simply removes synapses after damage has occurred.

Importantly, in mouse models of AD, the effects on synapses of key elements of AD pathogenesis—AβO, tau, or inflammation—are reversible. In multiple studies, deficits in LTP, memory impairment, and synapse loss recover in mice when levels of AβO, tau, or inflammation are lowered [69, 103–108]. This plasticity of synaptic connections and their potential for recovery lends hope for therapeutics that reduce synaptotoxicity in AD. Regardless of the causative role of AβO and the contributions to disease progression of tau, p-tau, glia, and inflammation processes, synapse dysfunction has a number of downstream neurophysiological consequences including altered neuronal oscillatory behavior and an imbalance between excitation and inhibition. These alter neural circuit function and adversely impact behavior. As such, normal synapse number and function is the basis for cognitive performance and is an ideal measure of brain damage due to disease.

## Biomarkers of synapse damage or loss

The importance of synapses in cognition and the strong links among synapses, AD pathophysiology, and the symptoms observed in AD make a compelling case for the use of biomarkers of synapse damage or loss as proxies for synaptic and cognitive function in AD. A recent publication of the NIA-AA Research Framework emphasized the necessity of a biological definition of the disease for clinical progress and established the A/T/N biomarker classification system, where “A” stands for amyloid beta, “T” for tau, and “N” for neurodegeneration [109], a broad concept that includes destruction of system-level circuits and regional volume loss, as well as injury to individual cellular elements such as axons, dendrites, and synapses. The extent to which this A/T/N biomarker classification system is confined to studies of the pathobiology of AD, versus used to define patient populations that are enrolled into clinical trials, will be subject of valuable scientific discussion [110]. In the remainder of this paper, we will focus on “N” type biomarkers specifically related to synapse damage or loss.

**Fig. 2:**
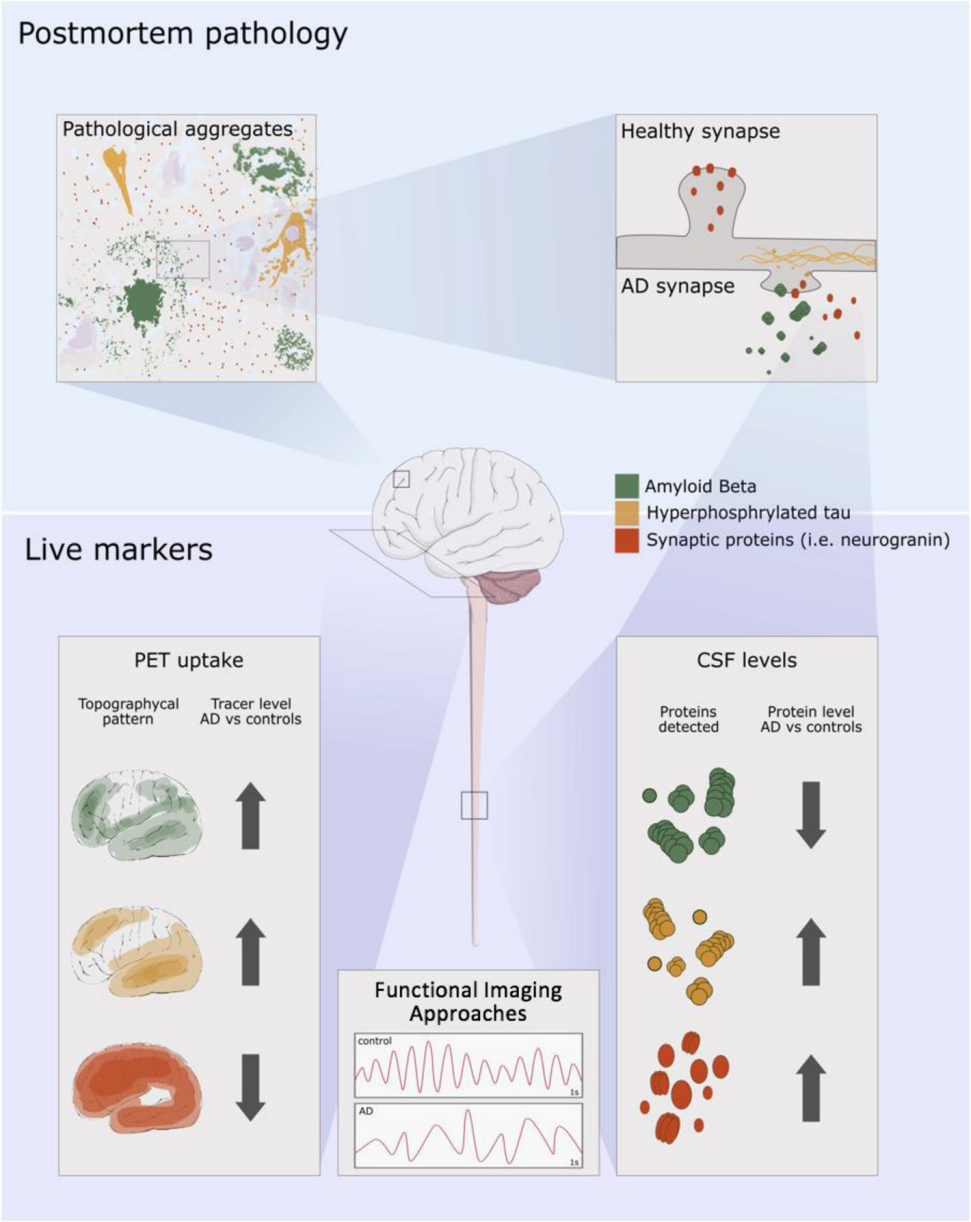
Amyloid and tau biomarkers can be used to confirm AD pathology, and biomarkers of synaptic damage and loss will be useful for predicting cognitive decline

Visualization of synapses in the living brain has recently been described through the labelling of synaptic vesicle glycoprotein 2A (SV2A) with the [^11^C]UCB-J positron emission tomography (PET) ligand [111–113]. (Additional SV2A radioligands, [^11^C]UCB-A and [^18^F]UCB-H, have also been under development.) Comparing a group of AD cases with cognitively healthy aged cases, a reduction of approximately 40% of SV2A signal was observed in the hippocampus in AD cases [114]. The use of this PET ligand to measure synapse loss longitudinally in AD is not yet well established. However, as a direct measure of synapse density, this biomarker in combination with other cerebrospinal fluid (CSF) biomarkers and functional imaging approaches, such as magnetic resonance imaging (MRI), quantitative electroencephalogram (qEEG), or fluorine-18 fluorodeoxyglucose PET (FDG-PET), is independent of the disease hypothesis and has the potential to be a strong indicator of brain degeneration and cognitive status (Fig. 2). Recent innovations such as this ability to sensitively measure synapse density in the brain of a living patient via SV2A PET imaging, low concentrations of synaptic protein proteolytic fragments in the CSF via sensitive ELISAs or LC/MSMS methods, changes in cortical synaptic currents measured by qEEG, or disruption of glucose metabolism measured by FDG-PET promise to revolutionize the ability to stage patients and to define disease more precisely. Furthermore, as synapses are a fundamental brain structure responsible for cognitive output, measures of synapse density have the most value in their ability to assess responses to disease-modifying treatments.

Following the identification of synaptic protein fragments of neurogranin, SNAP-25, and synaptotagmin in CSF [115, 116], specific protein biomarkers of synapse degeneration have begun to emerge in recent years. Protein fragments of neurogranin, a dendritic protein involved in LTP, are increased in CSF of patients with AD, and full-length neurogranin is decreased in post-mortem brain tissues [117, 118]. Furthermore, encouraging data show that increased neurogranin fragments in CSF correlate with future cognitive decline, brain atrophy, and glucose metabolism, even at early stages of the disease [117, 119–121], and that the increase in CSF neurogranin seems to be specific for AD [122, 123]. This use of CSF measurement of neurogranin concentration underlies the concept that an accurate biomarker of synapse loss reflects cognitive function based on the correlation between cognitive function and synaptic proteins in the post-mortem brain.

Other synaptic proteins including SNAP25, RAB3A, GAP43, AMPA receptor subunits, and a number of other proteins also show promise as CSF biomarkers of synaptic damage and loss [24, 25, 124, 125]. In addition, recent research proposes inflammatory markers, detectable in the CSF, as possible biomarkers of neurodegeneration in AD, though their correlation to synapse loss in particular remains unclear [126]. Biomarkers of glial activation such as CSF TREM2, chitotriosidase, CCL2, and YLK-40 have been observed in AD CSF [127–130]. Eventually, a panel of synaptic protein biomarkers may be a reliable readout for the different aspects of synapse loss (pre-synaptic, synaptic vesicle, and dendritic) and a predictor of memory decline. Indeed, a recent study found that a group of synaptic proteins changes in CSF before markers of neurodegeneration are observed in AD [131]. Although CSF collection is more invasive than blood sampling, robust blood-based biomarkers of synaptic damage are not yet available. It is, for example, possible to measure neurogranin concentration in plasma, but there is no plasma-CSF correlation [119, 132]. It may be possible to develop higher sensitivity assays and analyses of neuron-derived exosomes in blood with advancing technologies [130, 133].

Finally, functional imaging approaches are additional tools for visualizing the health and function of neurons affected by AD. EEG represents a dynamic measurement of synaptic function in cortical pyramidal neuronal dendrites that can capture the summed excitatory and inhibitory post-synaptic potentials at a macroscopic spatial scale with millisecond time resolution [134–137]. Overall, quantitative EEG analysis provides the most direct and dynamic *clinical* representation of neuronal and synaptic function in AD patients; however, while it is sensitive to changes in neuronal circuit responses resulting from synaptic dysfunction, it cannot discriminate between the exact mechanisms of action underlying synaptic dys/function. Alterations in quantitative measures derived from EEG data in patients with AD have been widely described and have been shown to be sensitive to disease progression [134, 138, 139] and to correlate with CSF biomarkers of AD [140]. Furthermore, EEG is non-invasive, robust, efficacious, and widely available in hospitals. Although EEG itself is an “old” technique, quantitative instead of visual analysis of EEG signals provides a wealth of information and is a novel and rapidly developing method in modern neuroscience. Spectral power measures (i.e., the percentage of the total brain activity accounted for by a specific wave frequency) in task-free EEGs can be calculated and reflect the oscillatory activity of the underlying brain network responsible for cognitive functioning. In patients with AD, the EEG shows distinct changes in spectral power indicating a gradual, diffuse slowing of brain electrical activity with progression of the disease [138]. In particular, the gradual relative increase of neuronal theta (4–8 Hz) activity appears to be a robust sign in early AD. It has been recently demonstrated that theta band activity is a marker of future cognitive decline in non-demented amyloid-positive subjects with additive value above other markers of disease progression such as medial temporal atrophy on MRI [141] and importantly that its increase can be reversed in response to approved AD therapeutics [142–151].

In addition to EEG, the use of fluorine-18 fluorodeoxyglucose PET (FDG-PET), which enables the visualization of glucose metabolism rates in the brain, has been investigated for its use in AD. In neurons, the demand for glucose is driven partly by synaptic terminals, which generate ATP needed for synthesis, release, and recycling of neurotransmitter molecules, for the maintenance of the normal resting potential and for the recovery from action potentials. The cerebral metabolic rate of glucose as measured with FDG-PET provides a direct index of synaptic functioning and an indirect measure of synaptic density [152]. Therefore, a disruption in glucose metabolism may be a very direct determinant of synaptic dysfunction [reviewed in [153]]. The ability to detect changes in glucose metabolism prior to the onset of clinical symptoms of AD may aid earlier diagnosis of AD [153]. Data from the Alzheimer’s Disease Neuroimaging Initiative (ADNI) have confirmed longitudinal associations between FDG-PET and clinical measures [154] and have suggested that FDG-PET may help to increase the statistical power of diagnosis over conventional cognitive measures, aid subject selection, and substantially reduce the sample size required for clinical trials [155, 156], though these findings must be confirmed in broader sample sizes and longer studies, and require further clarification regarding their applicability to AD or other types of dementias. Therapeutic trials have provided strong support for the use of FDG-PET as a clinically relevant primary biomarker outcome in proof of concept studies that has the power to detect active-placebo differences less than half as great as the best clinical measures [157]. However, additional studies showing a relationship between an effective treatment’s FDG-PET and clinical findings are needed to provide further support for its “theragnostic” value.

A key further issue for future exploration is the longitudinal relationship between biomarkers and cognitive outcome measures. Even modest correlations between the two would yield helpful evidence of clinical relevance. Recent studies have observed modest correlations between the International Shopping List Test, a measure of episodic memory, with various volumetric MRI measures and especially hippocampal volume [158]. Change over time correlations would provide further helpful support. Furthermore, as understanding of these biomarkers improves, their use may help in discerning AD from other types of dementias, in particular through localization of compromised synapses to the frontal lobe, temporal lobe, and other brain regions. Finally, opportunities for biomarker validation are offered by the extension of assessment to domains of cognition known to be compromised early in the disease process. Recent FDA draft guidance has called for trials to feature the use of “sensitive neuropsychological measures.” Commentators on the draft guidance have highlighted the need for trials to include measures of spatial memory skills, working memory, attention, and executive function [159].

## Conclusions

Synapses are essential parts of neurons that form the requisite connections of the neuronal networks that underlie cognition. The cognitive impairment in AD closely parallels the loss of synapses due to the toxic effects of Aβ, tau, and inflammation. Emerging biomarkers of synapse damage reflect such synapse injury and loss in the brain due to disease. Hence, biomarkers of synapse damage and loss, especially the use of multiple categories of biomarkers in combination with one another, hold great promise as biological measures that should correlate with cognitive function in AD.

## Data Availability

The datasets used and/or analyzed during the current study are available from the corresponding author on reasonable request.

## Abbreviations

AD: Alzheimer’s disease
APOE ε4: Apolipoprotein E epsilon 4
AβO: Aβ oligomers
CSF: Cerebrospinal fluid
EEG: Electroencephalography
FDA: US Food and Drug Administration
FDG-PET: Fluorine-18 fluorodeoxyglucose PET
LTP: Long-term potentiation
MMSE: Mini-Mental Status Examination
MRI: Magnetic resonance imaging
qEEG: Quantitative electroencephalography
SV2A PET: Synaptic vesicle glycoprotein 2A positron emission tomography
